# Plasma branched-chain amino acids and risk of radiation-induced acute skin toxicity in women with breast cancer: results from the ATHENA project

**DOI:** 10.3389/fonc.2025.1653293

**Published:** 2025-10-20

**Authors:** Sukshma Sharma, Francesca Bracone, Augusto Di Castelnuovo, Emilia Ruggiero, Amalia De Curtis, Chiara Cerletti, Giovanni de Gaetano, Francesco Deodato, Gabriella Macchia, Mariangela Boccardi, Savino Cilla, Alessio Giuseppe Morganti, Fulvio Mattivi, Andrea Anesi, Katia Petroni, Chiara Tonelli, Maria Benedetta Donati, Licia Iacoviello, Marialaura Bonaccio

**Affiliations:** ^1^ Research Unit of Epidemiology and Prevention, IRCCS Istituto Neurologico Mediterraneo Neuromed, Pozzilli, Italy; ^2^ Istituto di Radiologia, Università Cattolica S. Cuore, Rome, Italy; ^3^ Radiotherapy Unit, Responsible Research Hospital, Campobasso, Italy; ^4^ Medical Physic Unit, Responsible Research Hospital, Campobasso, Italy; ^5^ Radiation Oncology, IRCCS Azienda Ospedaliero-Universitaria di Bologna, Bologna, Italy; ^6^ Metabolomic Unit, Research Innovation Centre, Fondazione Edmund Mach, San Michele all’Adige, Italy; ^7^ Department of Biosciences, Università degli Studi di Milano, Milano, Italy; ^8^ Department of Medicine and Surgery, LUM University, Casamassima, Italy

**Keywords:** breast cancer, protein, radiotherapy, skin toxicity, branched-chain amino acid

## Abstract

**Background/Objectives:**

Little is known regarding the influence of circulating plasma branched-chain amino acids (BCAAs) such as leucine, isoleucine, and valine on acute skin toxicity (AST) after breast cancer (BC) radiotherapy. Hence, this study examined the association between circulating plasma BCAAs and the risk of ≥ grade 2 AST post-radiotherapy among BC patients.

**Methods:**

An observational study was conducted among 161 BC patients treated with radiotherapy within the ATHENA project in Italy. Plasma BCAAs were measured at 2-time points: at baseline (T0) and at the end of radiotherapy (T1) (after 3 or 5 weeks), and were ascertained using a validated method based on tandem mass spectrometry. AST was measured at T1 and defined according to the Radiation Therapy Oncology Group/European Organization for Research and Treatment Cancer (RTOG/EORTC) criteria. Analysis was conducted in two parts with separate study designs using multivariable-adjusted logistic regression models: 1) A cross-sectional analysis explored the association between plasma BCAAs at T1 and odds of AST post-radiotherapy; 2) A prospective analysis examined the association between plasma BCAAs at T0 and odds of AST post-radiotherapy.

**Results:**

AST post-radiotherapy was observed in 45 (28%) patients. In the cross-sectional analysis, at T1, plasma isoleucine (1-SD increment) was associated with 43% reduced odds of ≥ grade 2 AST post-radiotherapy (OR = 0.57;95% CI 0.36 to 0.91). A similar trend was observed in the prospective analysis at T0 (OR = 0.65;95% CI 0.42 to 1.02). There was no evidence of an association between plasma leucine and valine with AST post-radiotherapy, either at T0 or T1. Plasma isoleucine was associated with lower odds of AST post-radiotherapy in BC patients.

**Conclusions:**

The findings highlight that plasma isoleucine is associated with a low risk of ≥ grade 2 AST post-radiotherapy among BC patients. However, further studies such as isoleucine supplementation trials are needed to validate these findings.

## 1 Introduction

Breast cancer (BC) is the most commonly diagnosed type of cancer in women, with an estimated 2.3 million new cases in 2022, thus posing a major burden on public health (1). Surgery, chemotherapy, and radiotherapy are the most common modalities for BC treatment. Among these, radiotherapy remains a highly cost-effective single mode of treatment, accounting for only 5% of the overall cancer care costs. It is estimated that approximately 80% of BC patients must receive radiotherapy at some point in their treatment, hence highlighting its essential role in BC recovery (2, 3). Although widely used, radiotherapy also damages healthy tissues in the irradiation field. The patients mostly develop skin damage, and it is usually dependent on patient-related factors (age, hemoglobin levels, smoking habits, comorbidities including cardiovascular disease, diabetes mellitus, obesity), and the location and duration of the breast organ area exposed in the radiotherapy (4, 5).

Nearly 85-95% of patients with all types of cancer report different degrees of skin damage induced by radiotherapy, known as radiation-induced skin injury. These are of two types: (1) acute skin toxicity (AST) involving dry and wet desquamation and skin ulcers, and (2) chronic skin toxicity changes including chronic ulcers, keratosis due to radiation and fibrosis (6). In the Athena Study (N = 161), around 62% of patients experienced acute skin toxicity, according to a recent publication (7). Plasma-free amino acids are either ingested or endogenously synthesized, circulate abundantly, and are metabolic regulators in humans (8). Among them, branched-chain amino acids (BCAAs) such as leucine, isoleucine, and valine are essential amino acids that the body cannot synthesize and must be introduced through the diet to support healthy protein synthesis. Studies have suggested that red meat, fish, dairy products, and eggs are rich sources of BCAAs (9–12). The key functions of BCAAs are as follows: (1) activate the mammalian target of rapamycin (mTOR) signaling pathway required for protein synthesis, especially leucine; (2) enhance immunity and glucose consumption, especially isoleucine; (3) prevent cellular damage caused due to oxidative stress, especially valine; (4) insufficient or excess BCAAs levels enhance lipolysis (13–15).

Studies have suggested that both higher dietary and elevated circulating BCAAs levels were associated with a lower severity of BC (16, 17) by suppressing tumor growth and metastasis (17, 18). Interestingly, there is sparse evidence on the influence of amino acids, especially BCAAs, on the risk of radiation-induced skin injury in cancer, specifically BC.

However, it is not known whether BCAAs such as leucine, isoleucine, and valine influence the risk of AST after radiotherapy among BC women. Therefore, the main objective of this study was to examine the association between circulating plasma BCAA levels and the risk of ≥ grade (G) 2 (moderate/severe) AST among 161 BC patients’ post-radiotherapy within the ATHENA project in Italy.

## 2 Materials and methods

### 2.1 Trial design and participants

We used data from the ATHENA project for the current observational study. The ATHENA project was a double-blind, randomized, placebo-controlled trial designed to evaluate the impact of anthocyanin supplementation derived from purple corn cobs on radiation-induced skin toxicity in women with BC undergoing radiotherapy.

**Figure 1:**
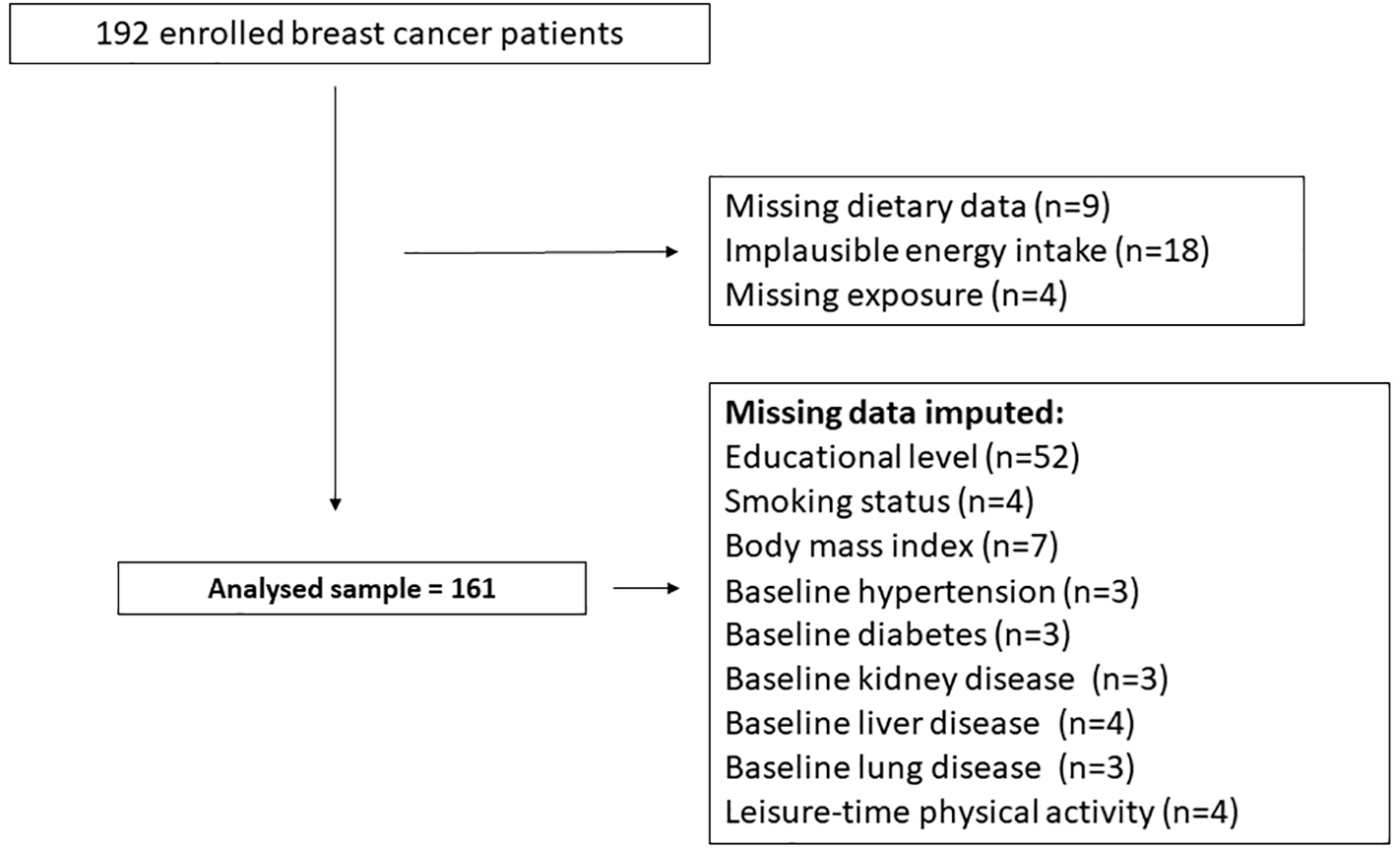
Flow chart of the ATHENA trial.

For the purposes of the present analyses, data from 161 women with BC who received radiotherapy were included. Comprehensive methodological details regarding the ATHENA randomized controlled trial (RCT) are available in previously published reports (19). Participants’ flow chart is provided in **Figure 1**.

### 2.2 Eligibility criteria

Women aged 18 years or older with a confirmed diagnosis of BC and deemed suitable for radiotherapy were considered for inclusion in the current analysis. Participant screening and selection were carried out by clinical staff based on inclusion and exclusion criteria established by the ATHENA RCT protocol (19).

Inclusion criteria encompassed patients with invasive breast carcinoma who had undergone breast-conserving surgery (lumpectomy or quadrantectomy) along with axillary staging procedures. Exclusion criteria included pregnancy or lactation at the time of recruitment, documented psychiatric or substance use disorders, as well as a history of non-invasive BC, synchronous bilateral invasive disease, non-epithelial breast tumors, multicentric carcinoma, or any prior radiotherapy to the breast or thoracic region for any indication (19). The ATHENA RCT was performed at the Gemelli Molise Hospital Radiotherapy Unit in Campobasso, Italy, and was conducted according to the guidelines of the Declaration of Helsinki.

Recruitment occurred between June 9, 2014, and June 26, 2017, with study follow-up concluding on October 10, 2018. The study received approval from the Ethics Committees of both the Catholic University of Rome and the Regional Health Authority of Molise (ASReM). All participants provided written informed consent. The trial was registered on ClinicalTrials.gov (Identifier: NCT02195960) and was conducted in compliance with Consolidated Standards of Reporting Trials (CONSORT) reporting guidelines (20).

### 2.3 Radiotherapy treatment

Within the ATHENA RCT, radiotherapy protocols were tailored based on individual recurrence risk profiles (low or moderate to high).

Participants classified as low risk underwent a hypofractionated radiotherapy regimen over a three-week period, receiving a total dose of 40 Gray (Gy) to the remaining breast tissue, with a simultaneous integrated boost of 4 Gy directed to the tumor bed. Those at moderate to high risk were administered a 5-week treatment with standard doses of radiotherapy, consisting of 50 Gy to the residual breast and a 10 Gy boost to the tumor bed.

All patients (3- and 5-week schedules) were treated with forward-planned intensity-modulated radiation therapy (IMRT) and were instructed to apply a topical cream (Atonderma Radiomed^®^) to the irradiated site approximately 2–3 hours before and after each session, starting from the first day of treatment (19).

For the present analysis, data from the entire cohort receiving radiotherapy were included, regardless of the specific treatment schedule or risk stratification.

### 2.4 Study exposure

#### 2.4.1 Determination of plasma branched-chain amino acids

Plasma BCAAs (leucine, isoleucine, and valine) were extracted and analyzed according to the protocol by Anesi et al. (21). Briefly, the plasma samples stored in the Neuromed Biobanking Centre were thawed on ice, and 25 µl aliquots were loaded onto Ostro plates (Waters, Milan, Italy) together with 25 µl of deuterated internal standards in methanol. Protein precipitation and metabolite extraction were achieved by loading 75 µl of ice-cold acetonitrile containing 1% formic acid; plates were covered and shaken on an orbital shaker for 5 min at 600 rpm. Subsequently, plates were filtered for 5 min using a positive pressure manifold with nitrogen at 4 psi. Extraction was repeated by adding 75 µl of ice-cold acetonitrile containing 1% formic acid. Filtrates were dried down using nitrogen at 37 °C and re-constituted in 200 µl of water containing 0.5% formic acid, and 1 mM ammonium formate. Samples were randomized prior to extraction. Quality control (QC) samples were created by pooling together 10 µl of each plasma sample and were extracted as described above. 2 µl of each sample were injected and analyzed by ultrahigh-performance liquid chromatography-tandem mass spectrometry (UHPLC-MS/MS) by using Multiple Reaction Monitoring (MRM) on a ABSciex 6500+ triple quadrupole connected to a Shimadzu LC-30 pump (ABSciex, Milan, Italy). Metabolites were separated on a Waters HSST3 column (100 x 2.1 mm, 1.8 µm) by using water 0.1% formic acid (A) and acetonitrile 0.1% formic acid (B) as mobile phases. Chromatographically resolved isoleucine (RT: 2.00 min) and leucine (RT: 2.20 min) were quantified by using MRM 132.1>86.2 in positive ion mode, valine (RT 1.20 min) by MRM 118.1>72.0. QC samples were injected at the beginning of the analytical sequence to condition UHPLC-MS/MS and at fixed intervals during the sequence to check the performance of our methodology. We ran 400–500 samples in one batch, each run lasting for approximately 10 minutes. QC was then injected at specific time points during acquisition to ensure stable instrument response (<20% indicated good stability throughout the acquisition). All the QC were prepared by pooling together equal amount of samples and were extracted in the same manner. Finally, samples were randomized prior to extraction. However, T0 and T1 samples from the same subject were acquired consecutively or vice versa to get the same MS response.

Finally, plasma BCAAs (leucine, isoleucine, and valine) were measured twice, at T0 (baseline) and again at T1 (after 3 or 5 weeks of undergoing radiotherapy).

### 2.5 Definition of study outcomes

The primary objective of this analysis was to assess the likelihood of experiencing AST greater than Grade 2. Skin reactions were evaluated by clinical staff during follow-up visits after completion of radiotherapy, using the RTOG/EORTC criteria for both acute and late toxicities (22).

AST was assessed at a follow-up time point (T1), corresponding to either 3- or 5-weeks post-treatment, depending on the radiotherapy schedule.

The irradiated area was examined for specific skin changes and reactions. Skin toxicity was dichotomized for analysis: a score of ‘0’ included Grade 0 (no visible skin changes) and Grade 1 (symptoms such as follicular, faint or dull erythema/epilation/dry desquamation/decreased sweating), while a score of ‘1’ included Grade 2 and above, indicating more pronounced effects such as tender or bright erythema, patchy moist desquamation/moderate edema (Grade 2); confluent, moist desquamation other than skin folds, pitting edema (Grade 3); and ulceration, hemorrhage, necrosis (Grade 4) (22, 23).

### 2.6 Assessment of covariates

At the baseline assessment (T0), data were collected on each participant’s medical history, anthropometric and clinical parameters, as well as dietary and lifestyle behaviors.

The following variables were included as covariates in the analysis: age (in years), body mass index (calculated as kilograms/meters square), C-reactive protein (CRP) levels at T0, total energy intake (calculated as kilocalorie [kcal] per day), education (0= none; 1= primary school diploma and secondary school diploma; 2= high school diploma; 3= bachelor’s degree and master’s degree and master/doctorate/post-doctorate), physical activity levels (0= no activity or sedentary; 1= moderate intensity; 2=vigorous-intensity), hypertension (systolic blood pressure [SBP] >140 mmHg and/or diastolic [DBP] >90 mmHg or anti-hypertensive treatment), smoking habits (1=yes, 0=no or 2=former), treatment classification (B=treatment [anthocyanin supplementation]/A=placebo) recorded at baseline (T0), and weeks of radiotherapy (3 or 5 weeks). The sensitivity analysis was adjusted for chemotherapy treatment (1=yes or 0=no).

We used directed acyclic graphs (DAGs) (24, 25) to identify the covariates to then include in the analyses as they provide a straightforward and visual presentation for identifying and testing assumptions about causal relationships between variables by deducing an algorithm, thus providing an adjustment set of covariates for estimating causal effects.

### 2.7 Statistical analyses

Analyses were performed in two parts: (1) a cross-sectional analysis to study the association between plasma BCAAs at T1 and the odds of ≥ G2 AST at T1 at the end of radiotherapy (3 or 5 weeks); (2) a prospective analysis for the association between plasma BCAAs at T0 and the odds of ≥ G2 AST at T1 at the end of radiotherapy (3 or 5 weeks).

Data for categorical variables are represented as numbers and percentages, and for continuous variables represented as mean and standard deviation (SD). For the association between plasma BCAAs and the odds of ≥ G2 AST amongst post-radiation BC patients, we used multivariable-adjusted logistic regression models to derive odds ratios (ORs) and corresponding 95% confidence intervals (CI).

For logistic regression, AST was coded as a binary outcome variable (‘0’ denoted the absence of ≥ G2 AST, and ‘0’ denoted the presence of ≥ G2 AST). Plasma BCAAs (leucine, isoleucine, and valine) data were recorded as continuous variables, and for interpretation, the ORs were computed for 1-SD increments in plasma BCAAs.

Analyses were conducted constructing four models: (i) Model 1: unadjusted logistic regression presented for each plasma BCAA and ≥ G2 AST; (ii) Model 2: ORs separately presented for individual associations between each plasma BCAA and ≥ G2 AST, and minimally adjusted for age, BMI, weeks of radiotherapy (3 or 5 weeks) and treatment group (treatment/placebo); (iii) Model 3: Additionally adjusted for smoking habits, CRP-levels at baseline, education, physical activity levels, hypertension, and total energy intakes; (iv) Model 4: Mutually adjusted for other plasma BCAAs in the model, and fully adjusted for age, body mass index, CRP levels at baseline, smoking habits, total energy intake (kcal), education, physical activity level, hypertension, weeks of radiotherapy (3 or 5 weeks) and treatment classification (treatment/placebo).

In a sensitivity analysis, Model 4 was further adjusted for chemotherapy treatment (yes or no) to examine the robustness of associations.

Missing data on covariates are listed in **Figure 1**. To maximize data availability, missing data were handled using single imputation with the PROC MI procedure in SAS. A regression-based imputation method was employed to estimate the missing values.

For analysis, we used two-sided statistical tests, and the significance was set at 95% CI. We used STATA/SE software version 18.0 (StataCorp, College Station, TX, USA) and SAS/STAT software, Version 9.4.

## 3 Results

The sample size of the current study was 161 women with a mean age of 57 years (SD ± 10.3). Refer to **Table 1** for the characteristics of the ATHENA participants. Around 28% (n=45) of women reported AST ≥G2 of the total sample. 28.5% (n=46) were hypertensive, and 98% (n=158) reportedly used prescribed hormone therapy, particularly letrozole (42%, n=69). Further, based on the BMI (kg/m^2^), 43.4% (n=70) participants were overweight and 16.1% (n=26) as obese.

**Table 1:** Baseline characteristics of the participants in the ATHENA trial (N = 161).

Variables	Mean or frequency
N of participants (%)	161 (100)
Age, years (mean, SD)	57.0 (10.3)
Educational level, n (%)	
None	3 (1.8)
Up to lower secondary	34 (21.1)
High school	45 (28)
Upto postgraduate	27 (16.8)
Do not know	52 (32.3)
Body mass index, kg/m^2^ (mean, SD)	26.4 (4.4)
Body mass index (BMI) (kg/m^2^), n (%)	
18.5-24.9 (normal)	65 (40.3)
25-30 (overweight)	70 (43.6)
>30 (obese)	26 (16.1)
Co-morbidities, yes, n (%)	
Hypertension	46 (28.5)
Diabetes Mellitus	8 (5)
Physical activity levels, yes, n (%)	
Moderate intensity	21 (13)
Vigorous intensity	7 (4)
Smoking status, n (%)	
Yes	28 (17)
No	106 (66)
Former	27 (17)
Use of Hormone therapy, yes, n (%)	158 (98)
Use of letrozole, yes, n (%)	69 (42)
Chemotherapy, yes, n (%)	77 (48)
Antocyanin treatment, yes, n (%)	83 (52)
Acute skin toxicity, n (%)	
Grade 0	49 (30)
Grade 1	67 (42)
Grade 2	43 (27)
Grade 3	2 (1)
Duration of radiotherapy, n (%)	
3 weeks	56 (35)
5 Weeks	105 (65)
Menopause, yes, n (%)	100 (62)
Plasma branch-chained amino acids (BCAA) (µmol/mL), (mean, SD) (observed range: minimum-maximum)	
Baseline (T0)	
Valine (ref range: 150 to 310 µM)	351.9 (80.6) (167-627)
Isoleucine (ref range: 42 to 100 µM)	102.5 (25.7) (47-233)
Leucine (ref range: 66 to 170 µM)	175.1 (34.3) (97-320)
First visit (T1)	
Valine (ref range^a^: 150 to 310 µM)	370.2 (86.4) (157-600)
Isoleucine (ref range: 42 to 100 µM)	105.6 (26.0) (38-214)
Leucine (ref range: 66 to 170 µM)	182.2 (34.4) (85-287)

a The reference range for plasma BCAAs in a general population is from https://www.ucsfhealth.org/ (the University of California San Francisco).

Finally, the plasma BCAAs (leucine, isoleucine, and valine) measurements conducted at T0 and T1 remained mostly similar before and after radiotherapy (T0 and T1).

In the fully adjusted multivariable logistic regression model (Model 4) of the cross-sectional analysis (refer to **Table 2**), of the three plasma BCAAs measured at T1, plasma isoleucine (1-SD increment) was associated with 43% reduced odds of ≥ G2 AST post-radiotherapy (OR = 0.57; 95% CI 0.36 to 0.91; p = 0.01).

**Table 2:** Association between plasma branch chain amino acids (BCAA) levels and the odds of post-radiation ≥G2 acute skin toxicity in cross-sectional (T1) and longitudinal analysis (T0) in ATHENA project.

Plasma BCAA (1SD increment) N = 161	Odds of skin toxicity, Model 1			Odds of skin toxicity, Model 2			Odds of skin toxicity, Model 3			Odds of skin toxicity, Model 4		
Cross-sectional (T1)	OR^a^	95% CI	*p* value	OR^b^	95% CI	*p* value	OR^c^	95% CI	*p* value	OR^d^	95% CI	*p* value
Isoleucine	0.92	0.80 to 1.06	0.30	0.88	0.75 to 1.02	0.11	0.89	0.76 to 1.04	0.16	0.57	0.36 to 0.91	0.01
Leucine	0.98	0.89 to 1.09	0.84	0.96	0.86 to 1.07	0.51	0.96	0.86 to 1.08	0.59	1.36	0.89 to 2.08	0.14
Valine	0.99	0.95 to 1.03	0.82	0.98	0.93 to 1.02	0.42	0.98	0.94 to 1.03	0.60	1.01	0.89 to 1.15	0.84
	Odds of skin toxicity, Model 1			Odds of skin toxicity, Model 2			Odds of skin toxicity, Model 3			Odds of skin toxicity, Model 4		
Longitudinal (T0)	OR^a^	95% CI	*p* value	OR^b^	95% CI	*p* value	OR^c^	95% CI	*p* value	OR^d^	95% CI	*p* value
Isoleucine	0.97	0.85 to 1.12	0.75	0.95	0.82 to 1.09	0.50	0.94	0.79 to 1.10	0.47	0.65	0.42 to 1.02	0.06
Leucine	1.00	0.91 to 1.11	0.89	0.99	0.89 to 1.10	0.87	0.99	0.88 to 1.11	0.91	1.08	0.73 to 1.58	0.69
Valine	1.00	0.96 to 1.05	0.65	0.99	0.95 to 1.04	0.98	1.00	0.95 to 1.05	0.81	1.10	0.95 to 1.26	0.18

OR, odds ratio; CI, confidence intervals; CRP, C-reactive protein.

a Linear regression models presented for each plasma BCAA

b Odds separately presented for individual associations between each plasma BCAA and ≥ G2 skin toxicity, and minimally adjusted for age, body mass index,

treatment group (treatment/placebo), radiotherapy duration (3 or 5 weeks).

c Additionally adjusted for smoking habits, CRP levels at baseline, education, physical activity levels, hypertension, total energy intake (kcal),

and smoking habits (yes or no).

d Mutually adjusted for other plasma BCAAs in the model and fully adjusted for age, body mass index, CRP levels at baseline, smoking habits,

total energy intake (kcal), education, physical activity level, hypertension, smoking habits (yes or no), radiotherapy duration (3 or 5 weeks) and

treatment classification (treatment/placebo).

Further, in Model 4 of the prospective analysis, an inverse association was observed between plasma isoleucine (1-SD increment) and the odds of ≥G2 AST post-radiotherapy (OR = 0.65; 95% CI: 0.42 to 1.02; p = 0.06), although the estimate did not reach conventional levels of statistical significance and should be interpreted with caution due to limited precision. However, when valine and leucine BCAA were individually or together analyzed along with other covariates in the models, there was no evidence of an association with ≥ G2 AST post-radiotherapy (refer to Models 1–4 in **Table 2**). Finally, in a sensitivity analysis, the association between plasma isoleucine and odds of ≥ G2 AST post-radiotherapy resulted statistically significant after an additional adjustment for chemotherapy treatment (refer to **Table 3**).

**Table 3:** Sensitivity analysis: Association between plasma branch chain amino acids (BCAA) levels and the odds of post-radiation ≥G2 acute skin toxicity in cross-sectional (T1) and longitudinal analysis (T0) in ATHENA project.

Plasma BCAA (1SD increment) N = 161	Odds of skin toxicity		
Cross-sectional (T1)	OR^a^	95% CI	*p* value
Isoleucine	0.58	0.36 to 0.93	0.02
Leucine	1.35	0.88 to 2.06	0.16
Valine	1.01	0.89 to 1.15	0.84
	Odds of skin toxicity		
Longitudinal (T0)	OR^a^	95% CI	*p* value
Isoleucine	0.67	0.95 to 1.26	0.18
Leucine	1.05	0.70 to 1.58	0.78
Valine	1.10	0.95 to 1.26	0.18

OR, odds ratio; CI, confidence intervals; CRP, C-reactive protein.

a Mutually adjusted for other plasma BCAAs in the model and fully adjusted for age, body mass index, CRP levels at baseline, smoking habits, total energy intake (kcal), education, physical activity level, hypertension, smoking habits (yes or no), radiotherapy duration (3 or 5 weeks), treatment classification (treatment/placebo) and chemotherapy (yes/no).

## 4 Discussion

The current study examined the influence of plasma BCAAs (leucine, isoleucine, and valine) on post-radiation ≥ G2 AST in a BC cohort. Findings in the cross-sectional analysis indicated that among plasma BCAAs measured at T1, plasma isoleucine was associated with lower odds of ≥ G2 AST post-radiotherapy. Meanwhile, the prospective analysis findings suggested similar trend in the statistical significance for the results between plasma isoleucine measured at baseline (T0) and the odds of ≥ G2 AST post-radiotherapy. To the best of our knowledge, this is the first study investigating the association between circulating plasma BCAAs (leucine, isoleucine, and valine) and the odds of ≥ G2 AST amongst BC patients who underwent radiotherapy.

The association of isoleucine levels with lower odds of ≥ G2 AST after the radiotherapy at T1 could indicate the protective short-term effects of isoleucine after radiotherapy in BC patients. Indeed, radiotherapy triggers a series of inflammatory responses in the normal tissue. Oxidative stress causes cell injury, producing a reaction of lymphocytes and macrophages, subsequently releasing pro-inflammatory cytokines and fibroblast stimulation, releasing reactive oxygen species (ROS) (4).

A study explored isoleucine’s immunological response in relation to immunotherapy for tuberculosis and suggested that isoleucine was strongly correlated with beta-defensin, predominantly secreted from leukocytes and epithelial cells (26). To elaborate, beta-defensin are small peptides (15–20 residues) that have antimicrobial defense properties by penetrating the microbe’s cell membrane and causing microbial death in a method similar to antibiotics. Therefore, this mechanism could potentially be postulated in the context of AST post-radiotherapy in BC. However, further studies are warranted to precisely identify this effect.

In the past, few studies only suggested the use of glutamine (in enteral form) to improve wound matrix formation in patients with hypercatabolic conditions, including cancers requiring radiotherapy and its side effects, such as mucositis and radiodermitis (27–29). Further, in relation to radiation-induced skin injury in BC, glutamine-treated BC patients post-radiotherapy demonstrated a lower rate of AST as compared to the corresponding placebo group (9% developed grade I AST vs. 80% developed grade II AST)—based on the RTOG/EORTC criteria (22) for AST (29).

Additionally, previous nutritional studies evidenced that high-protein diets might lower the risk of AST but not the protective effect of circulating plasma BCAAs in the context of radiotherapy treatment-related skin injuries (6). In recent years, the potential role of dietary BCAAs has been explored in relation to the risk of BC. To elaborate, one animal model study explored the impact of BCAAs in their dietary form on the risk of breast metastasis among mice. The findings demonstrated that high BCAA concentration impaired the ability of tumor cells to invade and migrate due to the downregulation of N-cadherin (18). In contrast, another study examined the long-term dietary intakes of BCAA and reported no evidence of an association with invasive BC risk (30). However, interestingly, there is a dearth of evidence for BCAAs in relation to radiotherapy treatment-related side effects, such as AST affecting BC patients.

Our findings showed that plasma leucine and valine were not associated with lower odds of post-radiotherapy ≥ G2 AST in BC patients. This is despite leucine being the most abundant amino acid responsible for muscle repair and maintaining energy homeostasis. This could elucidate the complex interplay between isoleucine and leucine in mammalian epithelial cells, ultimately promoting healthy growth and proliferation of breast cells and increasing longevity. Moreover, in animal studies, both leucine and isoleucine are suggested to improve the fractional protein synthesis rates in bovine mammary glandular cells with phosphorylation of mTOR, a protein kinase responsible for immune response, autophagy, and maintenance of health cellular metabolism—protein degradation is lowered. However, the pathways involving valine are so far unknown (31–33).

From an oncology nutrition standpoint, the current findings could provide the groundwork for dietary BCAA administration for BC patients undergoing radiotherapy. BCAAs account for 30-40% of the essential amino acids and cannot be synthesized in the body. Therefore, they need to be supplied through the diet (34, 35). Moreover, BCAAs are suggested to act as a fuel source to slower protein degradation during catabolic diseases (9, 36). In addition, further studies including isoleucine supplementation trials could validate our study’s findings to increase overall clinical significance.

To compensate for the protein losses the following BCAAs-related clinical strategies could be considered: (1) the time of administration, especially BCAA supply (before or immediately after radiotherapy); and (2) the form of protein (a) BCAA-rich animal or vegetable sources (for example, fish, meat, eggs, and pulses and cereals such as soybean meal, wheat germ, rye, barley, and sorghum) (9–12, 37) and (b) dietary supplementation rich in BCAAs [commercial supplement formulations comprised of protein-rich substrates such as soybean or chicken breast] (38, 39).

Finally, the sensitivity analysis adjusted for chemotherapy resulted statistically significant for BC patients; both those who underwent radiotherapy and those who underwent chemotherapy coupled with radiotherapy (40).

### 4.1 Strengths and limitations

Our study had some strengths to be considered. The study design and the analysis used in the methodology optimized the study findings. We had the available data on the BC patients’ circulating plasma BCAAs and repeated post-radiation ≥ G2 AST measures recorded over different time points during the follow-up period. Plasma BCAAs were ascertained using a validated method based on tandem mass spectrometry, providing accurate measurements in plasma samples. The AST readings were classified according to the international guidelines (RTOG grading) to make the findings widely applicable and replicable in clinical settings. We note that the clinicians assessing AST were completely blinded to BCAA levels and study hypotheses, since plasma BCAAs were measured retrospectively from samples collected during routine care. This blinding supports the objectivity and strengthens the credibility of our findings. The regression models were carefully adjusted for covariates with possible clinical significance (24, 25). The analyses were adjusted for radiotherapy duration (3 or 5 weeks) and anthocyanin treatment/placebo, thus accounting for the original study design (19) and commonly used cancer treatments, such as chemotherapy, to test their robustness.

There were some limitations to be considered. First, the study had a modest sample size (N = 161), which could justify the lack of statistical associations, and part of the analysis was cross-sectional. Second, the cross-sectional findings captured relatively shorter time points. However, plasma BCAAs represent the real-time status of the BCAA circulation. They are available in abundant quantities sufficient to exhibit any immediate changes in the metabolism post-radiotherapy among women with BC. Therefore, tracking the plasma BCAAs status after each patient’s radiotherapy visit during radiotherapy treatment might assist the doctors in making clinical decisions, such as dietary supplementation. Third, there were no recommendations/guidelines for plasma BCAA reference ranges available for a population with breast cancer. Hence, it was challenging to compare the differences in plasma BCAAs during baseline and post-radiotherapy and make appropriate clinical conclusions. Fourth, we did not apply correction for multiple comparisons. This choice was motivated by the exploratory nature of the study and the relatively small sample size, which may limit statistical power. However, we acknowledge this as a limitation that could increase the likelihood of false-positive findings. Finally, we could not examine the association between dietary BCAAs and radiation-induced AST in BC patients because of the lack of available data. Perhaps future studies could explore the longitudinal associations between dietary BCAAs and the risk of AST in women with BC.

## 5 Conclusions

Among circulating plasma BCAAs, high plasma isoleucine was associated with lower odds of ≥ G2 AST among BC women who underwent radiotherapy. However, further studies such as isoleucine supplementation trials are needed to validate the protective role of isoleucine thus contributing to evidence-based clinical management strategies for BC women undergoing radiotherapy.

## Data Availability

The data underlying this article will be shared upon reasonable request to the corresponding author. The data are stored in an institutional repository (https://repository.neuromed.it) and access is restricted by the ethics approval and the legislation of the European Union.
